# Comparative analysis of selected bioactive components (fatty acids, tocopherols, xanthophyll, lycopene, phenols) and basic nutrients in raw and thermally processed camelina, sunflower, and flax seeds (*Camelina sativa* L. Crantz, *Helianthus* L., and *Linum* L.)

**DOI:** 10.1007/s13197-019-03899-z

**Published:** 2019-07-19

**Authors:** Bożena Kiczorowska, Wioletta Samolińska, Dariusz Andrejko, Piotr Kiczorowski, Zofia Antoszkiewicz, Malwina Zając, Anna Winiarska-Mieczan, Maciej Bąkowski

**Affiliations:** 1 Institute of Animal Nutrition and Bromatology, University of Life Sciences in Lublin, Akademicka Street 13, 20-950 Lublin, Poland; 2 Department of Biological Bases of Food and Feed Technologies, University of Life Sciences in Lublin, Głeboka Street 28, 20-612 Lublin, Poland; 3 Department of Animal Nutrition and Feed Science, University of Warmia and Mazury in Olsztyn, Oczapowskiego Street 5, 10-719 Olsztyn, Poland

**Keywords:** Oil seeds, Processed, Nutrients, Bioactive constituents

## Abstract

The aim of study was to determine the content of basic nutrients, the level of fatty acids, tocopherols, xanthophyll, and lycopene, and the total phenolic content in camelina (*Camelina sativa* L. Crantz) (Cs), sunflower (*Helianthus* L.) (Ha), and flax (*Linum* L.) (Lu) seeds. The seeds were either raw or subjected to processing, i.e. boiling, micronization, or microwave roasting. The basic chemical composition was established and the fatty acid composition as well as the content of tocopherol (α, β, γ, δ, total), β-carotenoids, xanthophyll, lycopene, and total phenolics were determined in the analyzed oil seeds. The analyzed oil seeds are a rich source of protein and PUFAs as well as α-tocopherols (Ha) and γ-tocopherols (Cs, Lu), xanthophyll, and phenolics One portion of seeds covered from 746/513 (Cs) to as much as 1209/813% (Lu) (female/male) of the ALA daily intake. The AI value in the processed seeds increased (*P* < 0.05) and the values of H/H and HC declined (*P* < 0.05). The oil seed processing resulted in loss of most nutrients and bioactive constituents and appearance of some amounts of *trans* isomers, especially in the microwave roasted seeds (0.99–1.79 g/100 g crude lipid). The phenolic content decreased in the boiled seeds (Ha: 1301; Cs: 578.3, and Lu: 62.75 mg/100 g).

## Introduction

Oil seeds, especially traditional ones, are an important component of the human diet in countries where they are part of the traditional nutrition model based primarily on vegetable products and in highly developed countries, where they are regarded as health-enhancing food. Although they are high in calories and contain large amounts of fat, oil seeds are rich in fatty acids that are indispensable for the normal function of the organism, e.g. n-3 unsaturated fatty acids, including α-linolenic acid (ALA) (Ghanbari et al. 2012). Additionally, oil seeds contain phytochemicals (e.g. phytosterols), natural antioxidants, and bioactive carbohydrates. When consumed in appropriate amounts, they can reduce the risk of cancer development. Therefore, oil seeds are regarded as desirable supplements of plant-poor diets and nutrition based on highly processed foods. In view of adoption of healthier nutritional regimes, it is recommended that consumption of oil seeds should increase in the Western diet (Shahidi and Ambigaipalan 2015; DeLuca et al. 2018).

Traditional seeds of oil-bearing plants such as sunflower, flax and, more recently, camelina are consumed as raw food in developing countries. In contrast, they are commonly used as an additive to bakery, cereal, and confectionery products, ready-made food, and sweet and savory snacks in highly developed regions. However, insufficient attention in the literature is paid to the impact of culinary thermal processes, both the traditional boiling and microwave treatments, which are commonly used in households, or industrial-scale treatments such as infrared irradiation (micronization). Thermal treatments used in the processing of high-fat vegetable raw materials usually lead to modifications of the chemical composition, e.g. losses of fats. There are also not always beneficial changes in the composition of fatty acids. Microwave roasting can lead to degradation of phenolic compounds, which is largely dependent on the type of raw material and process parameters (Juhaimi et al. 2018). On the other hand, microwave waves or evaporation of high-fat raw materials applied as pretreatment before fat extraction can have a positive effect the content of β-carotene and lycopene in the final products (Tuyen et al. 2013). Another aspect of cooking fat-rich foods was investigated by Groopman et al. (2015). They have found that cooking peanuts can increase the digestibility of lipids. The cooking process induced changes in the integrity of the cell walls in the high-fat raw materials, which made the lipid structures available to digestive lipases. Research conducted in a mouse model confirmed that cooked peanuts had increased calorie content.

Therefore, the aim of study was to determine the content of basic nutrients, the level of fatty acids, tocopherols, xanthophyll, and lycopene, and the total phenolic content in camelina (*Camelina sativa* L.Crantz), sunflower (*Helianthus* L.), and flax (*Linum* L.) seeds, which were either raw or subjected to culinary thermal processing, i.e. boiling, micronization, or microwave roasting.

## Materials and method

### Plant material

The consumer seeds of camelina (Cs), sunflower (Ha), and flax (Lu) used in the study were purchased (~ 5 kg of each) in a specialized store (Seed Centre, Lublin, Poland), which sells certified plant material originating from crop harvesting from a given year. The research material originated from cultivation areas located in southern and eastern Poland. The seeds originating from the 2015 and 2016 harvest were purchased in September of both years.. All seeds were processed at the same time to prevent changes in their chemical composition that could be induced by storage. The characteristics of the research material and the details of sample preparation are presented in Table 1. All seeds were boiled (B) in a 3-L vessel in 2 L of distilled water at 97 °C. Micronization (IR) was carried out twice at identical parameters. The seeds were placed on the tape in a single ca. 100-mm layer. An infrared ray generator with a 400-W ESC-1 infrared radiator and an average filament temperature of approximately 500 °C was used for the thermal processing. The infrared radiation wavelength was λ = 2.5–3 μm. The radiator was placed at a distance of ca. 100 mm from the seed layer. The microwave roasting (MWR) of the seeds was carried out twice in the same process conditions. The seeds placed in a single layer were roasted on 20-cm diam. Petri dishes.

**Table 1:** Common, scientific names, and procedure of oil seed preparation

Common English name	Camelina	Sunflower	Flax
Scientific name	*Camelina sativa* L. Crantz	*Helianthus annuus* L.	*Linum usitatissimum* L.
Cultivar	Luna	Lech	Opal
Common Polish name	Lnianka. rydz. lennica	Słonecznik	Siemię lniane
Weight processed (g)	500	500	500
Notes	Whole	Dehulled	Whole
Boiling time (min.)^a^	30	30	30
Micronization parameters	160 °C over 60 s	160 °C over 60 s	160 °C over 60 s
Microwave roasting parameters	2400 MHz over 15 min	2400 MHz over 15 min	2400 MHz over 15 min
*Treatment scheme*			
Raw seeds-control	Cs-R^b^	Ha-R^c^	Lu-R^c^
Boiled seeds	Cs-B^e^	Ha-B^f^	Lu-B^g^
Micronized seeds	Cs-IR^h^	Ha-IR^i^	Lu-IR^j^
Microwave roasted seeds	Cs-MWR^k^	Ha-MWR^l^	Lu-MWR^m^

^a^Boiling was performed in 2.0 L of tap water in all cases, ^b^raw camelina seed, ^c^raw sunflower seed, ^d^raw flax seed, ^e^boiled camelina seed, ^f^boiled sunflower seed, ^g^boiled flax seed, ^h^micronized camelina seed, ^i^micronized sunflower seed, ^j^micronized flax seed, ^k^microwave roasted camelina seed, ^l^microwave roasted sunflower seed, ^m^microwave roasted flax seed, results are mean ± standard deviation of three analyses

The cooked seeds were placed in a colander for 5 min to drain excess water. The raw, boiled, micronized (infrared radiation), and microwaved (microwave radiation) seeds were homogenized. A portion of homogenates served for determination of moisture and ash content. The other part was freeze-dried and sealed in plastic bags under vacuum and kept at − 20 °C until further analyses.

### Basic composition

A random sample was taken for chemical analysis. The content of dry matter and basic nutrients in ground seed samples (250 g seeds/variety) was determined according to standard AOAC procedures (2011). The content of total carbohydrates was calculated as dry matter–crude protein–crude lipid–crude ash. The energy value of the seeds is based on the Atwater general factors (UE Low 2011). The energy value of oil seeds expressed in kcal was converted into kJ with a coefficient of 4.1868.

### Fatty acids

The fatty acid composition was determined with the gas chromatography method on a Varian CP-3800 chromatograph CP-3800 (Varian Inc., Palo Alto, USA) after conversion of the fats to fatty acid methyl esters (FAME) according to the AOAC method (1990). The determinations were based on a template such as Supelco 37-Component Fame Mix (Sigma-Aldrich Poznan, Poland). Fatty acid data are presented as the percentage of total fatty acids in the seed sample (Wu et al. 2007) The levels of *trans* isomers in the processed oil seeds were determined using the same apparatus with an SPTM-2560 capillary column (Supelco Inc., Bellafonte, PA, USA), The determinations were based on such templates as Supelco 37-Component FAME MIX and Linolenic Acid Methyl Ester Isomer Mix.

The saturated fatty acid/unsaturated fatty acid (S/P), atherogenic (AI) and thrombogenic (TI) indexes (Ulbricht and Southgate 1991), and the hypocholesterolemic/hypercholesterolemic ratio (HH) (Santos-Silva et al. 2002) were calculated in all analyzed seeds.

The unsaturation index (UI) was calculated, with *trans* fatty acids considered as saturated acids (Geiser et al. 1994).

The content of the vitamin E (CE) (Eittenmiller et al. 1988) and the Harris coefficient (HC) equivalent was calculated for the oil seeds (Witting 1972). The content of SFA, linolenic acid, ALA, and *trans* fatty acids (FA) per 100 g of crude lipid was converted into their content in one serving of raw and processed oil seeds with the use of the 0.956 conversion factor and the lipid content of the samples (Greenfield and Southgate 2003).

### Tocopherols

Tocopherols were isolated from the freeze-dried samples by extracting 0.5 g of the sample with 55 mL hexane containing 20 mg/L DYN. The simple solid–liquid extraction procedure for the quantitative analysis of the vitamin E content in nuts has been reported to be superior to other extraction procedures (Delgado-Zamarreno et al. 2001). Aliquots of 20 µL of solutions were subjected to reversed-phase high-performance liquid-chromatography (RP-HPLC) in an HPLC system (Agilent Technologies, Model 1050, Waldbronn, Germany) with a quaternary pump and UV (280 nm, 295 nm) and fluorescence (kex = 295 nm, kem = 330 nm) detection. External standard quantification was performed based on a series of five different standard concentrations of α-, γ-, and δ-tocopherol.

### Xanthophyll (Carotenoids)

Samples were injected using a 100 µL-loop on a JASCO Autosampler (model AS-950-10; JASCO, Tokyo, Japan) onto a C18 RP (Vydac 201TP45; Bucher Biotec, Basel, Switzerland) column. The samples were eluted isocratically in the HPLC mobile phase at a flow rate of 1.2 mL/min (Agilent Technologies, Model 1050, Waldbronn, Germany), a multiwave programmable detector (model MD 910, JASCO), and a Borwin PDA version 1.50 system controller (JASCO). Carotenoids were identified and quantified at 450 nm against known standards.

### Total phenolic content

The total phenolic content was determined using the spectrophotometric method (Singleton and Rossi 1965). A methanolic solution of the extract at a concentration of 1 mg/mL was used in the analysis. The determinations were carried out using the Folin–Ciocalteu reagent. The results were expressed as GAE (Gallic Acid Equivalents).

### Statistical analysis

The analyses were performed in triplicate and all data were expressed as means and standard deviation. The percentage data of fatty acids were arcsine transformed. The normality of data and homogeneity of variances were tested with the Shapiro–Wilk and Brown–Forsythe tests, respectively. The data were analyzed statistically using the species as an independent variable of one-way ANOVA or non-parametric Kruskal–Wallis test All statements of significance were based on the 0.05 and 0.01 probability levels (Statistica version 13).

## Results and discussion

### Basic nutrients and energy content

The oil seeds exposed to infrared and microwave radiation were characterized by a higher (*P* < 0.05) concentration of nutrients in the dry matter, with the exception of crude fiber (Cs-IR, Cs-MWR, Ha-IR, Ha-MWR, Lu-IR, and Lu-MWR) (Table 2). Thermal treatments applied to plant raw materials without the use of water, i.e. micronization (IR) or microwave radiation (MWR), are associated with substantial product drying. During thermal processing of plant raw materials without an external moisture factor, unbound water present in plant tissues is removed. This leads to contraction of the raw material, and the changes in the water content alter the concentration of the cell sap. The thickening of the dry matter components leads to a change in the density, viscosity, and osmotic pressure in the raw material. This, in turn, may lead to destruction of cytoplasmic membranes and changes in their physical and chemical properties (Biswas et al. 2007; Kiczorowska et al. 2016). Boiling was the only treatment that reduced the dry matter content in the analyzed camelina seeds from 874 g/kg d.m to 753 g/kg d.m. (*P* < 0.05). Reduction of the moisture content in vegetable food products by thermal processes increases their microbiological safety and extends their shelf life.

**Table 2:** Nutrient composition and energy content in raw and processed seeds (g/kg)

Seeds	Dry matter		Crude ash		Crude protein^a^		Crude lipid		Crude fiber		Available carbohydrates^b^		Energy^c^ (kcal)		Energy^c^ (kJ)	
Camelina																
Cs-R^d^	874	± 0.54	41.8	± 0.64	254.3	± 0.15	383.4	± 0.23	110.7	± 0.18	83.8	± 0.24	5024	± 0.56	21036	± 0.41
Cs-B^e^	753	± 0.34	37.4	± 0.21	176.2	± 0.27	359.1	± 0.34	103.4	± 0.26	76.9	± 0.39	4451	± 0.34	18636	± 0.37
Cs-IR^f^	956	± 0.26	40.2	± 0.38	192.7	± 0.31	406.6	± 0.18	74.3	± 0.24	242.2	± 0.26	5548	± 0.27	23227	± 0.19
Cs-MWR^g^	978	± 0.21	40.6	± 0.43	183.4	± 0.42	387.4	± 0.27	83.5	± 0.39	283.1	± 0.19	5520	± 0.18	23109	± 0.15
*P*-value^h^																
Cs-R versus Cs^i^	0.048		0.153		0.026		0.237		0.034		0.015		< 0.01		0.031	
Sunflower																
Ha-R^j^	849	± 0.31	36.1	± 0.48	244.5	± 0.23	529.3	± 0.19	21.4	± 0.34	17.7	± 0.41	5855	± 0.15	24515	± 0.35
Ha-B^k^	768	± 0.41	33.5	± 0.34	154.3	± 0.17	516.7	± 0.12	19.8	± 0.28	43.7	± 0.32	5482	± 0.23	22952	± 0.24
Ha-IR^l^	982	± 0.19	34.2	± 0.21	196.4	± 0.19	536.2	± 0.26	16.4	± 0.16	198.8	± 0.28	6439	± 0.37	26960	± 0.16
Ha-MWR^m^	991	± 0.27	35.1	± 0.19	187.3	± 0.34	521.9	± 0.34	17.3	± 0.37	229.4	± 0.16	6399	± 0.21	26789	± 0.28
*P*-value^h^																
Ha-R versus Ha^i^	0.185		0.226		0.029		0.194		0.037		0.013		0.097		< 0.01	
Flax																
Lu-R^n^	930	± 0.41	36.4	± 0.33	211.3	± 0.26	436.3	± 0.34	73.5	± 0.23	172.7	± 0.24	5610	± 0.19	23487	± 0.34
Lu-B^o^	864	± 0.32	35.9	± 0.26	194.6	± 0.19	409.6	± 0.61	64.3	± 0.12	159.6	± 0.39	5232	± 0.17	21905	± 0.19
Lu-IR^p^	973	± 0.29	35.7	± 0.19	220.5	± 0.34	425.2	± 0.28	39.8	± 0.36	251.8	± 0.26	5796	± 0.21	24265	± 0.28
Lu-MWR^r^	986	± 0.17	36.1	± 0.18	215.7	± 0.42	419.7	± 0.19	47.1	± 0.45	267.4	± 0.17	5804	± 0.35	24300	± 0.37
*P*-value^h^																
Lu-R versus Lu^i^	0.264		0.183		0.345		0.197		0.029		0.043		0.264		0.318	

^a^Calculated by Kjeldhal nitrogen N × 6.25. ^b^Calculated by the difference (dry matter–crude protein–crude lipid–crude ash–crude fiber). ^c^In 100 g dry matter, ^d^ raw camelina seed, ^e^ boiled camelina seed, ^f^micronized camelina seed, ^g^microwave roasted camelina seed, ^h^*P* < 0.05, 0.01 statistical differences, ^i^raw seeds compared to processed seeds: boiling (B), micronized (IR), microwave roasted (MWR), ^j^raw sunflower seed, ^k^boiled sunflower seed, ^l^micronized sunflower seed, ^m^microwave roasted sunflower seed, ^n^raw flax seed, ^o^boiled flax seed, ^p^micronized flax seed, ^r^microwave roasted flax seed

The traditional and modern methods for oil seed processing did not induce changes in the total content of mineral compounds, i.e. crude ash. Similarly, there were no significant changes in the content of crude lipid. However, literature provides evidence that the action of a thermal factor on vegetable food products can result in fat loss (Kiczorowska et al. 2016). This is associated with the increased activity of free radicals that are capable of recombination. The phenomenon occurs at high temperature generated during the boiling process or inside the tissue structures of a raw material penetrated by infrared and microwave radiation. This may lead to formation of lipid-starch complexes i.e. the so-called resistant starch, which can be detected in the fat fraction. This may seem to indicate lack of an impact of the factors used on this fraction. In turn, the treatments exerted an effect on the total protein content in the camelina, sunflower, and flax seeds. The decline in the protein amount (*P* < 0.05) was intensified in the boiled seeds. The camelina (Cs-B) and sunflower (Ha-B) seeds proved to be especially susceptible to the boiling process, as their protein content decreased by approximately 31 and 37%, in comparison with Cs-R and Ha-R. Micronization induced the lowest (*P* < 0.05) loss of total protein in the camelina (Cs-IR) and sunflower (HA-IR) seeds, in comparison with the raw seeds (Cs-R and Ha-R) (approximately 24.2 and 19.7%, respectively). The total protein in the flax seeds exhibited the lowest degree of degradation induced by the processing treatments. Changes in the protein fraction are mainly related to loss of amino acids, in particular lysine, methionine, and cysteine, which are extremely sensitive to high temperatures. The processing is associated with formation of hardly digestible protein-fat complexes as well as melanoidins, i.e. volatile Maillard reaction compounds responsible for e.g. the smell and color of products. These include methional formed from methionine and converted very easily into volatile reactive sulfur compounds (methanethiol, dimethyl disulfide). However, the aroma of roasted seeds is desirable and required by consumers. Changes in proteins induced by high temperature involve their physical properties, e.g. solubility, which is particularly important in digestion processes. The temperature of heat treatments of plant raw materials should not exceed 80 °C to maintain the thermal stability and solubility of proteins. In the case of flax seeds, thermal treatment can even increase the digestibility of this raw material by removal of excessive amounts of mucilage (Marambe et al. 2013). The high digestibility of micronized raw plant materials has been confirmed in animal studies by Kiczorowska et al. (2015a, b).

All the culinary oil seed treatments analyzed in this study caused a decrease (*P* < 0.05) in the content of crude fiber and an increase (*P* < 0.05) in total carbohydrates. The greatest changes were induced in the flax seeds. The lowest level of fiber components (*P* < 0.05) was detected in the micronized (ca. 32.9% in Cs-IR, 23.4% in Ha-IR, and 45.8% in Lu-IR) and microwave roasted seeds, in comparison with Cs-R, Ha-R, and Lu-R (ca. 24.6% in Cs-MWR, 19.1% in Ha-MWR, and 35.9% in Lu-MWR). The reduction in the total fiber content is associated with the increasing solubility of hardly digestible fractions, which may be reflected to some extent in an increased amount of carbohydrates. This may lead to formation of new anhydroglucose bonds via additional transglucosidation (Kiczorowska et al. 2016). In highly processed vegetable raw materials, cellulose and hemicellulose polysaccharides are degraded to simple sugars, which are easily digested in the gastrointestinal tract (Kiczorowska et al. 2015a, b). Simultaneously, the product loses its fibrous properties and does not serve a ballast function (Elleuch et al. 2011). Nevertheless, high content of the soluble fiber fraction in the diet is beneficial. It contributes to reduction of blood cholesterol and triacylglycerol levels by binding cholesterol supplied with food in a gel structure. This decreases cholesterol absorption and increases excretion thereof in the feces.

The changes in the chemical composition induced in Cs-R, Ha-R, and Lu-R by boiling, micronization, and microwave processing resulted in reduction of their calorific value. The processed camelina seeds (Cs-B) proved to have the highest dietary value, as their energy value declined by 11.4%, compared with Cs-R. The calorific value of the sunflower and flax seeds did not change significantly (*P* < 0.05). Nour et al. (2015) reported similar reduction in the energy value of boiled sorghum seeds.

### Fatty acids and lipid parameters

#### Saturated and unsaturated fatty acids

Although no significant modifications were found for the content of crude lipid, there were changes in its qualitative composition (*P* < 0.01, *P* < 0.05). In the case of high-fat raw materials, there are inevitable changes in the fat fraction during thermal processes. Especially the roasting or irradiation processes exert a substantial effect on the fat fraction structure. Irradiation of plant raw materials increases the temperature inside tissue structures. In such conditions, free radicals increase the recombination activity to form substances that can react with other components. Lipids at the level of fatty acids can form stable complexes with sugar and protein compounds. These complexes can be determined in the crude fat fraction, which may suggest that no such changes occur in raw fat (Kiczorowska et al. 2016). Only a thorough analysis of the fatty acid composition indicates the trend and intensity of these modifications. The fatty acid profile of the oil seeds subjected to the culinary treatments showed saturation of acids accompanied by an increase (*P* < 0.05) in the saturated fatty acid (SFA) content (Table 3) at the expense of the unsaturated fatty acid (UFA) fraction (camelina, *P* < 0.05) (Table 3). The changes in the SFA profile in the processed camelina, sunflower, and flax seeds (*P* < 0.05) were mainly influenced by the levels of palmitic and stearic acids, which represent the highest proportion in the total fatty acid content, i.e. 5.15–5.90 and 2.62–4.47 g/100 g of crude lipid, respectively (Table 3). A significant effect of boiling (B) as well as infrared (IR) and microwave (MWR) radiation was also found in the case of the content of margaric acid (Cs, Ha; *P* < 0.05), arachidic acid (Cs, Lu; *P* < 0.01 and S; *P* < 0.05), and behenic acid (Cs, Ha; *P* < 0.05) in the crude lipid. However, their proportion in the total fatty acid content is low; hence, they exert an inconsiderable effect on the total fatty acid pool in the analyzed seeds. The highest effect on the changes in the SFA fraction (*P* < 0.05) was exerted by the infrared and microwave processes. The camelina and flax seeds appeared to be particularly sensitive to these thermal treatments, as they exhibited 19–21% and 24–26% higher SFA amounts than those determined in Cs-R and Lu-R (Cs-IR–Cs-MWR and Lu-IR–Lu-MWR, respectively). Similar results were reported by Kiczorowska et al. (2015a, b) in cereal grains processed at high temperatures.

**Table 3:** Saturated and unsaturated fat acid content in raw and processed seeds (g/100 g crude lipid)

Seeds	Saturated fat acids																						
	C14:0		C16:0		C17:0		C18:0			C20:0			C21:0			C22:0		C24:0			SFA^a^		
Camelina																							
Cs-R^b^	0.06	± 0.37	5.41	± 0.18	0.04	< 0.01	2.62	± 0.78		1.13	± 0.18		1.61	± 0.27		0.27	± 0.08	–		–	11.14		± 0.34
Cs-B^c^	0.04	± 0.54	5.45	± 0.34	0.02	< 0.01	3.66	± 0.24		1.15	± 0.24		1.59	± 0.19		0.24	± 0.16	–		–	12.15		± 0.28
Cs-IR^d^	0.05	± 0.21	6.58	± 0.28	0.03	< 0.01	3.64	± 0.19		1.18	± 0.31		1.55	± 0.34		0.25	± 0.14	–		–	13.28		± 0.41
Cs-MWR^e^	0.05	± 0.19	6.56	± 0.71	0.03	< 0.01	3.73	± 0.34		1.23	± 0.46		1.63	± 0.56		0.21	± 0.17	–		–	13.44		± 0.16
*P*-value^f^																							
Cs-R versus Cs^g^	0.264		0.037		0.045		0.043			< 0.01			0.305			0.036		–			0.019		
Sunflower																							
Ha-R^h^	0.07	± 0.28	5.90	± 0.36	0.05	< 0.01	3.83	± 0.24		0.17	± 0.09		–	–		0.64	± 0.18	–		–	10.66		± 0.16
Ha-B^i^	0.05	± 0.16	6.78	± 0.18	0.03	< 0.01	4.54	± 0.19		0.16	± 0.01		–	–		0.51	± 0.26	–		–	12.07		± 0.28
Ha-IR^j^	0.02	± 0.34	6.89	± 0.24	0.04	< 0.01	4.67	± 0.34		0.19	± 0.03		–	–		0.35	± 0.34	–		–	12.16		± 0.31
Ha-MWR^k^	0.03	± 0.73	6.71	± 0.21	0.03	< 0.01	3.98	± 0.26		0.21	± 0.08		–	–		0.39	± 0.11	–		–	11.35		± 0.46
*P*-value^f^																							
Ha-R versus Ha^g^	0.029		0.038		0.049		0.025			0.023			–			0.031		–			0.034		
Flax																							
Lu-R^l^	0.04	< 0.01	5.15	± 0.41	0.06	< 0.01	4.47	± 0.19	0.15		± 0.03		0.05	< 0.01		–	–	0.01		< 0.01	9.93		± 0.38
Lu–B^m^	0.04	< 0.01	6.26	± 0.35	0.06	< 0.01	5.49	± 0.34	0.13		± 0.06		0.04	< 0.01		–	–	0.01		< 0.01	12.03		± 0.24
Lu-IR^n^	0.04	< 0.01	6.52	± 0.26	0.07	< 0.01	5.52	± 0.16	0.10		± 0.02		0.05	< 0.01		–	–	0.01		< 0.01	12.31		± 0.16
Lu-MWR^o^	0.03	< 0.01	6.64	± 0.17	0.06	< 0.01	5.63	± 0.25	0.14		± 0.11		0.04	< 0.01		–	–	< 0.01		–	12.55		± 0.17
*P*-value^f^																							
Lu-R versus Lu^g^	–		0.043		–		0.036		< 0.01			0.263			–				–			0.044	

Seeds	Unsaturated fat acids																			
	C16:1		C18:1 *n-9*		C18:2 *n-6*		C18:3 *n-3*		C20:1 *n-9*		C20:4 *n-6*		C22 :1		C22:6 *n-3*		MUFA^p^		PUFA^r^	
Camelina																				
Cs-R^b^	0.10	± 0.08	14.93	± 0.28	17.53	± 0.38	35.78	± 0.24	15.49	± 0.35	2.06	± 0.14	2.85	± 0.45	0.12	± 0.03	33.37	± 0.37	55.49	± 0.28
Cs-B^c^	0.09	± 0.04	14.86	± 0.16	16.73	± 0.12	35.97	± 0.19	15.19	± 0.19	2.05	± 0.23	2.85	± 0.29	0.11	± 0.06	32.99	± 0.19	54.86	± 0.16
Cs-IR^d^	0.08	± 0.01	15.28	± 0.37	16.34	± 0.26	34.82	± 0.52	15.16	± 0.24	2.01	± 0.31	2.90	± 0.17	0.13	± 0.09	33.42	± 0.24	53.30	± 0.37
Cs-MWR^e^	0.07	± 0.03	15.31	± 0.46	16.21	± 0.61	33.93	± 0.31	15.77	± 0.31	2.09	± 0.65	3.06	± 0.34	0.14	± 0.08	34.21	± 0.34	52.37	± 0.18
*P*-value^f^																				
Cs-R versus Cs^g^	0.128		0.264		< 0.01		0.136		0.347		0.183		0.251		0.094		0.128		0.034	
Sunflower																				
Ha-R^h^	0.08	± 0.02	27.22	± 0.27	61.68	± 0.31	0.11	± 0.03	0.14	± 0.06	0.01	< 0.01	0.1	± 0.77	–	–	27.54	± 0.18	61.80	± 0.27
Ha-B^i^	0.05	± 0.01	27.83	± 0.16	59.72	± 0.63	0.09	< 0.01	0.13	± 0.04	0.01	< 0.01	0.1	± 0.64	–	–	28.11	± 0.19	59.82	± 0.24
Ha-IR^i^	0.07	± 0.04	28.71	± 0.18	58.83	± 0.54	0.06	< 0.01	0.15	± 0.03	0.01	< 0.01	< 0.01	–	–	–	28.94	± 0.42	58.90	± 0.36
Ha-MWR^k^	0.06	± 0.03	29.46	± 0.34	58.91	± 0.16	0.07	< 0.01	0.13	± 0.02	0.01	< 0.01	< 0.01	–	–	–	29.66	± 0.32	58.99	± 0.62
*P*-value^f^																				
Ha-R versus Ha^g^	0.263		0.024		< 0.01		0.039		0.089		–		–		–		0.026		0.123	
Flax																				
Lu-R^l^	0.07	± 0.05	18.19	± 0.31	13.58	± 0.15	57.93	± 0.25	0.14	± 0.09	–	–	0.02	< 0.01	0.14	± 0.06	18.42	± 0.16	71.65	± 0.34
Lu-B^m^	0.06	± 0.04	17.33	± 0.28	14.61	± 0.34	55.72	± 0.36	0.12	± 0.05	–	–	< 0.01	–	0.12	± 0.03	18.52	± 0.34	70.45	± 0.26
Lu-IR^n^	0.07	± 0.02	16.74	± 0.26	14.68	± 0.29	55.99	± 0.18	0.09	± 0.07	–	–	0.01	< 0.01	0.11	± 0.09	17.91	± 0.28	70.78	± 0.27
Lu-MWR^o^	0.05	± 0.02	17.03	± 0.16	14.15	± 0.26	56.01	± 0.42	0.07	± 0.04	–	–	< 0.01	–	0.13	± 0.02	18.16	± 0.19	70.29	± 0.16
*P*-value^f^																				
Lu-R versus Lu^g^	0.138		0.034		0.047		< 0.01		0.025		–		–		0.139		0.154		0.268	

Results are mean ± standard deviation of three analyses. ^a^saturated fatty acid, ^b^raw camelina seed, ^c^boiled camelina seed, ^d^micronized camelina seed, ^e^microwave roasted camelina seed, ^f^*P* < 0.05, 0.01 statistical differences, ^g^raw seeds compared to processed seeds: boiling (B), micronized (IR), microwave roasted (MWR), ^h^raw sunflower seed, ^i^boiled sunflower seed, ^j^micronized sunflower seed, ^k^microwave roasted sunflower seed, ^l^raw flax seed, ^m^boiled flax seed, ^n^micronized flax seed, ^o^microwave roasted flax seed, ^p^monounsaturated fatty acids, ^r^polyunsaturated fatty acids

The methods employed for the seed treatment contributed to an increase (Ha, *P* < 0.05) in the content of monounsaturated fatty acids (MUFA) and a decline (*P* < 0.05) in the content of polyunsaturated fatty acids (PUFA) in the crude lipid (Table 3). The greatest changes in the MUFA level (*P* < 0.05) of processed oil seeds were induced by oleic acid, whose modifications were multidirectional and probably depended on the physico-chemical properties of the raw material. The sunflower seeds treated with infrared (Ha-IR) and microwave (Ha-MWR) radiation exhibited an even 5.5–8% increase (*P* < 0.05) in the oleic acid content (Ha-MWR), compared with the Ha-R treatment. In turn, this thermal treatment of the flax seeds (Lu-IR and Lu-MWR) contributed to loss (*P* < 0.05) of this acid (by 8–6% in comparison to Lu-R). The greatest changes in the PUFA pool were noted in the case of linoleic and linolenic acids in the camelina and flax seeds. The infrared and microwave radiation processes applied to the seeds induced the greatest changes (Cs-IR, Cs-MWR; *P* < 0.01; Lu-IR; *P* < 0.05) in the PUFA composition. As in the case of oleic acid, the direction and intensity of the changes in the content of linoleic and linolenic acids in the processed seeds varied. The content of these acids was reduced by approximately 7.5% (C18:2, Cs-MWR; *P* < 0.01), and even by 45% (C18:3 Ha-IR; *P* < 0.05).

Culinary thermal treatments of seeds may induce formation of dimers and cyclic compounds in fat structures. The main mechanism involved in this process is the homolytic cleavage of the C–C bond in position α or ß into a double bond and formation of radical molecules. Direct linkage of these radicals can lead to formation of short- and long-chain acids, dicarboxylic acids, and hydrocarbons. Radical elements can also bind hydrogen from another molecule of a fatty acid, usually oleic acid, leading to the formation of alkyl radicals. These, in turn, undergo dismutation to mono- and diene acids or bind to molecules of another fatty acid. Linoleic acid undergoes similar reactions as well. These reactions most often lead to the formation of a mixture of acyclic, bicyclic, and tricyclic dimers with a varied unsaturation degree, which can induce changes in the physico-chemical properties of fat (Biswas et al. 2007).

Although the culinary processing methods used in the study resulted in a decline in the content of total *n*-*6* fatty acids in the camelina and sunflower seeds (*P* < 0.01), the n-6/n-3 ratio, TI, and UI were unchanged (Table 4). In contrast, the AI value in the analyzed seeds increased (*P* < 0.05) and the values of HH and HC declined (*P* < 0.05 and *P* < 0.05, 0.01, respectively) under the influence of the investigated processes. The greatest changes in the fatty acid indices were induced by the micronization and microwave roasting treatments. The flax seeds exhibited the greatest changes.

**Table 4:** Lipid parameters in raw and processed seeds (g/100 g crude lipid)

Seeds	Total *n-6*		Total *n-3*		*n-6/n-3* ^*a*^		*Trans*		S/P^b^		AI^c^		TI^d^		HH^e^		UI^f^		HC^g^	
Cs-R^h^	19.59	± 0.37	35.90	± 0.46	0.55	± 0.25	0.00	–	0.13	± 0.08	0.064	< 0.01	55.55	± 0.51	12.87	± 0.16	175.8	± 0.36	0.51	± 0.19
Cs-B^i^	18.78	± 0.28	36.08	± 0.24	0.52	± 0.18	0.95	± 0.14	0.14	± 0.06	0.064	< 0.01	54.93	± 0.24	12.70	± 0.23	174.4	± 0.61	0.53	± 0.34
Cs-IR^j^	18.35	± 0.19	34.95	± 0.35	0.53	± 0.14	1.07	± 0.26	0.15	± 0.09	0.078	< 0.01	53.38	± 0.36	10.34	± 0.61	170.6	± 0.48	0.46	± 0.18
Cs-MWR^k^	18.30	± 0.52	34.05	± 0.16	0.51	± 0.17	1.25	± 0.28	0.16	± 0.01	0.076	< 0.01	54.45	± 0.19	10.54	± 0.34	174.4	± 0.16	0.48	± 0.21
*P*-value^l^																				
Cs-R versus Cs^m^	< 0.01		0.264		0.348		0.038		0.028		0.049		0.187		0.046		0.348		< 0.01	
Sunflower																				
Ha-R^n^	61.69	± 0.26	0.11	± 0.07	560.8	± 0.48	0.00	–	0.12	± 0.08	0.069	< 0.01	62.02	± 0.18	14.91	± 0.19	151.2	± 0.37	0.34	± 0.13
Ha-B^o^	59.73	± 0.18	0.09	± 0.04	663.7	± 0.34	1.37	± 0.24	0.14	± 0.07	0.079	< 0.01	60.08	± 0.32	12.83	± 0.37	147.8	± 0.24	0.22	± 0.18
Ha-IR^p^	58.84	± 0.27	0.06	± 0.08	980.7	± 0.16	1.20	± 0.36	0.14	± 0.04	0.079	< 0.01	59.16	± 0.16	12.68	± 0.46	146.8	± 0.18	0.26	± 0.13
Ha-MWR^r^	58.92	± 0.64	0.07	± 0.02	841.7	± 0.28	1.79	± 0.19	0.13	± 0.09	0.077	< 0.01	59.23	± 0.27	13.12	± 0.25	147.7	± 0.16	0.25	± 0.21
*P*-value^l^																				
Ha-R versus Ha^m^	< 0.01		0.057		0.246		0.046		0.097		0.037		0.136		0.031		0.276		0.029	
Flax																				
Lu-R^s^	13.58	± 0.34	58.07	± 0.48	0.23	± 0.07	0.00	–	0.11	± 0.01	0.059	< 0.01	71.70	± 0.28	17.31	± 0.21	219.4	± 0.39	0.26	± 0.18
Lu-B^t^	14.61	± 0.28	55.84	± 0.61	0.26	± 0.14	0.44	± 0.28	0.13	± 0.07	0.072	< 0.01	70.51	± 0.31	13.93	± 0.38	213.9	± 0.27	0.24	± 0.06
Lu-IR^u^	14.68	± 0.15	56.10	± 0.21	0.26	± 0.11	0.67	± 0.16	0.14	± 0.08	0.075	< 0.01	70.85	± 0.16	13.34	± 0.42	214.2	± 0.16	0.14	± 0.03
Lu-MWR^w^	14.15	± 0.23	56.14	± 0.38	0.25	± 0.05	0.99	± 0.47	0.14	± 0.09	0.076	< 0.01	70.36	± 0.12	13.09	± 0.67	213.5	± 0.24	0.17	± 0.05
*P*-value^l^																				
Lu-R versus Lu^m^	0.147		0.284		0.089		0.035		0.054		0.033		0.297		0.041		0.347		0.039	

Results are mean ± standard deviation of three analyses. ^a^in the PUFA *n*-*6*/PUFA *n*-*3* ratio, ^b^saturated fatty acids/unsaturated fatty acid, ^c^atherogenic index, ^d^thrombogenic index, ^e^hypocholesterolemic/hypercholesterolemic ratio, ^f^unsaturation index, ^g^Harris coefficient, ^h^raw camelina seed, ^i^boiled camelina seed, ^j^micronized camelina seed, ^k^microwave roasted camelina seed, ^l^*P* < 0.05, 0.01 statistical differences, ^m^raw seeds compared to processed seeds: boiling (B), micronized (IR), microwave roasted (MWR), ^n^raw sunflower seedm ^o^boiled sunflower seed, ^p^micronized sunflower seed, ^r^microwave roasted sunflower seed, ^s^raw flax seed, ^t^ boiled flax seed, ^u^micronized flax seed, ^w^microwave roasted flax seed

The values of the fatty acid indices calculated for the analyzed seeds indicate a need for substantial variation and, hence, optimization of the diet. It is important from the dietary point of view to maintain the n-6/n-3 acid ratio at a level from 4:1 to 2:1. An important component of n-3 acids is ALA, which is converted into DHA and EPA in the organism. With the high content of ALA, flax seeds are recommended as an element of nutritional prophylaxis e.g. in cardiovascular diseases and for menopausal females (DeLuca et al. 2018). However, the present investigations indicate high sensitivity of these seeds to the modification of the fat fraction and its health-promoting parameters induced by the thermal treatment. The health-enhancing conversion of ALA into DHA and EPA is dependent on e.g. the nutritional regime. It is blocked by an excessive level of consumed n-6 acids and *trans* fatty acids.

#### *Trans* fatty acids

All the thermal treatment methods employed in the study had an impact (*P* < 0.05) on the formation of *trans* isomers (Table 4). The sunflower seeds (Ha-B, Ha-MWR) turned out to be the most susceptible to the changes in the position of functional groups induced by the thermal factor, whereas the flax seeds exhibited the lowest susceptibility. In the Lu-B, Lu-IRM, and Lu-MWR treatments, the level of *trans* isomers was by half lower than that in the processed camelina and sunflower seeds.

Ha and Seo (2006) have reported that this type of transformation of fatty acids and triglycerides in processed food is mainly observed in unsaturated 18-carbon acids. Increased temperature and duration of the interaction can enhance the intensity of these changes. The transition of fatty acids into *trans* forms involves changes in their biological value and their effect on the organism. The incorporation of *trans* UFA into cell membrane phospholipids in place of acids with the *cis* configuration changes the properties of the membrane, i.e. its fluidity and permeability, the number and activity of receptors, and associated enzymes, which may promote development of diseases (Ferreri et al. 2002).

### Tocopherols

Determination of the tocopherol content in food products included in the daily diet is highly important for calculation of its daily intake. This ensures implementation of appropriate health-promoting dietary prophylaxis. Tocopherols are regarded as the most effective natural antioxidants in the lipid phase. They prevent lipid peroxidation by scavenging peroxide radicals that terminate chain reactions in membranes and lipoprotein particles (Traber and Atkinson 2007). In the present study, the content of tocopherols in the processed oil seeds was relatively variable, as it ranged from 159.6 (Ha-R) to 311.6 (Lu-R) and 412.2 μg/g (Cs-R) (Table 5). γ-Tocopherol predominated in the camelina and flax seeds, (α + γ)-tocopherols was dominant in the sunflower seeds, and β-tocopherol was the least abundant in all cases. Each of the analyzed seed processing methods reduced (*P* < 0.05) the tocopherol content, in particular that of α-tocopherol, by 35, 43, and 51.6%, in comparison with the Cs-R, Ha-R, and Lu-R treatments (Cs-MWR, Ha-B, and LU-IR,) respectively. As in the case of the other lipid fractions, the highest reduction of the tocopherol content was noted in the flax seeds. In terms of the culinary method, the highest reduction of tocopherol levels in the oil seeds was induced by micronization (Cs-IR, Ha-IR, Lu-IR) and microwave radiation (Cs-MWR, Ha-MWR, Lu-MWR).

**Table 5:** Tocopherols, carotenoids, and phenolics in raw and processed seeds

Seeds	Tocopherols (μg/g)										Carotenoids (μg/g)						Phenolics (mg/100 g)	
	α		β		γ		δ		Total		β		Xanthophyll		Lycopene		Total	
Camelina																		
Cs-R^a^	1.78	± 0.71	2.09	± 0.34	405.4	± 0.27	2.96	± 0.15	412.2	± 0.24	0.28	± 0.08	40.7	± 0.27	0.04	< .01	989.8	± 0.38
Cs-B^b^	1.56	± 0.54	1.98	± 0.24	387.2	± 0.34	2.56	± 0.24	409.1	± 0.33	0.26	± 0.04	40.2	± 0.32	0.03	< 0.01	578.3	± 0.24
Cs-IR^c^	1.17	± 0.29	2.05	± 0.16	375.7	± 0.51	2.30	± 0.31	380.5	± 0.30	0.27	± 0.04	39.8	± 0.19	0.03	< 0.01	653.7	± 0.16
Cs-MWR^d^	1.16	± 0.13	1.87	± 0.18	379.1	± 0.16	2.16	± 0.16	379.6	± 0.21	0.25	± 0.09	40.3	± 0.16	0.04	< 0.01	546.3	± 0.31
*P*-value^e^																		
Cs-R versus Cs^f^	0.038		0.264		0.089		0.175		0.206		0.314		0.197		0.248		0.023	
Sunflower																		
Ha-R^g^	93.9	± 0.27	0.59	± 0.19	64.7	± 0.27	21.63	± 0.27	159.6	± 0.24	0.24	± 0.04	36.8	± 0.36	0.0	–	2124	± 0.37
Ha-B^h^	53.1	± 0.34	0.46	± 0.24	63.5	± 0.18	20.89	± 0.48	160.2	± 0.31	0.18	± 0.08	31.6	± 0.24	0.0	–	1301	± 0.24
Ha-IR^i^	64.7	± 0.51	0.51	± 0.31	62.1	± 0.34	18.05	± 0.21	145.3	± 0.28	0.16	± 0.06	27.0	± 0.16	0.0	–	1578	± 0.19
Ha-MWR^j^	62.3	± 0.18	0.43	± 0.21	59.7	± 0.61	16.71	± 0.34	137.2	± 0.17	0.17	± 0.17	29.4	± 0.78	0.0	–	1947	± 0.43
*P*-value^e^																		
Ha-R versus Ha^f^	0.037		0.136		0.264		0.075		0.026		0.043		0.164		–		0.048	
Flax																		
Lu-R^k^	6.26	± 0.36	1.07	± 0.34	302.0	± 0.18	2.26	± 0.27	311.6	± 0.37	0.52	± 0.36	27.2	± 0.36	0.0	–	87.45	± 0.47
Lu-B^l^	4.56	± 0.61	0.89	± 0.26	256.2	± 0.24	2.09	± 0.16	298.4	± 0.42	0.48	± 0.27	20.1	± 0.25	0.0	–	62.75	± 0.15
Lu-IR^m^	3.03	± 0.27	0.61	± 0.18	159.1	± 0.37	1.15	± 0.24	163.8	± 0.19	0.29	± 0.19	16.8	± 0.41	0.0	–	79.84	± 0.39
Lu-MWR^n^	3.16	± 0.19	0.57	± 0.16	184.3	± 0.16	1.34	± 0.38	156.9	± 0.24	0.27	± 0.14	15.6	± 0.18	0.0	–	67.12	± 0.24
*P*-value^e^																		
Lu-R versus Lu^f^	0.029		0.037		0.043		0.027		0.031		0.046		0.039		–		0.037	

Results are mean ± standard deviation of three analyses, ^a^raw camelina seed, ^b^ boiled camelina seed, ^c^micronized camelina seed, ^d^ microwave roasted camelina seed, ^e^*P* < 0.05, 0.01. statistical differences, ^f^raw seeds compared to processed seeds: boiling (B), micronized (IR), microwave roasted (MWR), ^g^raw sunflower seed, ^h^boiled sunflower seed, ^i^micronized sunflower seed, ^j^microwave roasted sunflower seed, ^k^raw flax seed, ^l^boiled flax seed, ^m^micronized flax seed, ^n^microwave roasted flax seed

Tocopherols protect polyunsaturated fatty acids (PUFAs) contained in vegetable raw materials from oxidative processes by removal of biochemical and biophysical reactions of active oxygen species and free radicals through interruption of the lipid oxidation chain reaction. Alpha- and gamma-tocopherols are the most active forms. The thermal factor operating during infrared and microwave radiation or cooking induces processes leading to oxidation of PUFAs and activation of tocopherols resulting in their loss. Investigations conducted by Brenes et al. (2002) demonstrated that such a distribution was obviously a result of oil seed processing, which caused significant loss of tocopherols during common domestic processing of virgin olive oil, including frying, microwave heating, and boiling in water in a pressure cooker. Tocopherols are added to high-fat raw materials in the food industry to protect fat. However, their natural antioxidant activity is closely related to their content in fat and correlates with their concentration to a certain limit. Exceeding the limit may accelerate the oxidation process (Kmiecik et al. 2015). Investigations have indicated greater activity and efficiency of tocopherols occurring naturally in the raw material than those added to the product. Interesting research results were obtained by Hoed et al. (2017), who roasted pumpkin seeds at a temperature of 60 to 150 °C and did not observe a downward trend in the content of tocopherols with the increasing temperature of the process.

### Carotenoids and total phenolic content

The xanthophyll content ranged from 27.2 μg/g (Lu-R) to 40.7 μg/g (Cs-R) (Table 5). In turn, the level of β-carotene in the raw oil seeds ranged from 0.24 to 0.28 μg/g (Ha-R and Cu-R) to 0.52 μg/g (Lu-R). Trace amounts of lycopene, i.e. less than 0.04 μg/g, were only detected in the camelina seeds. The thermal processing techniques did not exert an effect on the content of all carotene compounds determined in the camelina seeds and xanthophyll in the sunflower seeds. Carotenoids in the flax seeds turned out to be the most sensitive compounds, as the loss of β-carotene was estimated at up to 29% (Ha-MWR) and 48% (Lu-MWR) and the decline in xanthophyll reached approximately 42.6% (Lu-MWR), in comparison with unprocessed seeds. Similar results were reported by Beta and Hwang (2018), who investigated orange maize flour subjected to heat and moisture treatment. The authors emphasize a significant correlation between the longer heating time and the higher moisture content in their effect on degradation of carotenoids. The literature provides data on the high thermostability of some carotenoids, in particular lycopene and β-carotene (Gumul et al. 2005; Nebesny and Budryn 2003). The authors demonstrate that even high-temperature processes, e.g. sterilization or cooking, do not cause large losses of these ingredients or a decrease in their antioxidant activities.

The total phenolic content in the crude oil seeds ranged from 87.45 mg/100 g (Lu-R) to 989.8 (Cs-R) and 2124 mg/100 g (Ha-R) (Table 5). These values were higher (*P* < 0.05) than those determined for the processed seeds. The lowest amounts of total phenolic acids were detected in the boiled flax (Lu-B, ca. 28%) and sunflower (Ha-B, ca. 39%) seeds and in the microwave roasted camelina seeds (Cs-MWR, ca. 45%) in comparison with the raw seeds (Lu-R, Ha-R, Cs-R). Imbibition exhibited by the oil seeds during the boiling process may have led to partial leaching, thermal degradation, and oxidation of these compounds. Similar findings were reported by Xu and Chang (2008), who observed a significant decrease in the total phenol content after soaking and boiling legume seeds. An opposite phenomenon in roasted pistachio nuts was observed by Rodríguez-Bencomo et al. (2015). The roasting treatment was accompanied by an increase in the total polyphenol content, but not in all phenolic compounds.

### Dietary intake via consumption of one serving of cooked oil seeds

The dietary intake of several macro- and micronutrients via consumption of one serving of oil seeds (24 g of camelina and flax and 46 g of sunflower seeds) was calculated and listed in Table 6. The results demonstrate that the consumption of one serving of raw oil seeds provides an intake of basic nutrients in the range of approximately 11–21% (female) and 9–17% (male) of daily protein (Cs-R–Lu-R, Ha-R), 0.5–2.9% of minimum daily dietary carbohydrates (Ha-R–Lu-R), 12–30% (female) and 10–26% (male) of daily fat (Cs-R–Ha-R), and 3–8.3% (female) and 2.5–6.8% (male) of daily fiber (Ha-R–Cs-R). Oil seeds provide relatively high amounts of calories, with one portion covering 6.4–13.5% (female) and 5.3–11.2% (male) of daily energy demand (Cs-R–Lu-R, Ha-R). In turn, they introduce low levels of SFA into the diet, which is consistent with the current dietary recommendations (USDA 2015). Additionally, they provide small amounts of xanthophyll, in comparison with the dose of 8 mg recommended as a prophylactic level protecting against eye diseases (Abdel-Aal et al. 2013). In the case of linoleic acid, one portion of camelina and flax seeds covers approximately 25% of the recommended intake, whereas one portion of sunflower seeds has a nearly 7-9-fold higher content of this acid. Camelina and flax seeds are valuable dietary components, as they are rich in ALA. One portion covers from 746/513 (Cs-R) to as much as 1209/813% (Lu-R) (female/male) of the daily intake. In turn, the amount of phenolics contained in one portion of raw oil seeds is highly diverse. Their daily intake can be covered in 12–475% (on average 243%) by camelina seeds, 49–1954% (on average 523%) by sunflower seeds, and 1–42% by and flax seeds (on average 21%), in comparison with 50 and 2000 mg provided by plant-rich and plant-poor diets (Sikora et al. 2008; USDA 2015).

**Table 6:** Percent coverage of daily supply for selected macro and micronutrients by consuming one serving^a^ of raw and processed seeds

Seeds	Protein	Carbohydrates	Lipid	Energy	Dietary fiber	SFA^b^	Linoleic acid^b^	ALA^b^	*Trans* FA^b^	Tocopherols	Xanthophyll	Phenolics
Camelina (1 tbsp)												
Cs-R^c^	5.33	1.76	8.04	120.6	2.32	0.86	4.02	8.21	0.00	9.89	0.98	237.6
Cs-B^d^	3.18	1.39	6.49	106.8	1.87	0.75	3.84	8.25	0.01	9.82	0.96	138.8
Cs-IR^e^	4.42	5.56	9.33	133.2	1.70	1.18	3.75	7.99	0.02	9.13	0.96	156.9
Cs-MWR^f^	4.30	6.64	9.09	132.5	1.96	1.17	3.72	7.78	0.03	9.11	0.97	131.1
Sunflower (1 cup)												
Ha-R^g^	9.55	0.69	20.67	269.3	0.84	2.11	27.12	0.05	0.00	7.34	1.69	977.0
Ha-B^h^	5.45	1.54	18.25	252.2	0.70	2.11	26.26	0.04	0.12	7.37	1.45	598.5
Ha-IR^i^	8.87	8.98	24.22	296.2	0.74	2.82	25.87	0.03	0.10	6.68	1.24	725.9
Ha-MWR^j^	8.54	10.46	23.79	294.4	0.79	2.58	25.91	0.03	0.19	6.31	1.35	895.6
Flax (1 tbsp)												
Lu-R^k^	4.72	3.85	9.74	134.6	1.64	0.92	3.12	13.3	0.00	7.48	0.65	21.0
Lu-B^l^	4.04	3.31	8.49	125.6	1.33	0.98	3.35	12.8	0.01	7.16	0.48	16.1
Lu-IR^m^	5.15	5.88	9.93	139.1	0.93	1.17	3.37	12.8	0.01	3.93	0.40	14.4
Lu-MWR^n^	5.10	6.33	9.93	139.3	1.11	1.19	3.25	12.9	0.02	3.77	0.37	15.1
Daily intake	46/56 g^o^	Minimum 130 g^c^	67/80 g^o^	2000/2400 kcal^o^	28/34 g^o^	< 22–27 g^od^	12–17 g^ost^	1.1–1.6 g^or^	1.2–6.7g^u^	10 mg^p^	8 mg^w^	50–2000 mg^z^

^a^One serving: 1 tbsp (tablespoon) = 24 g, 1 cup = 46 g, ^b^fatty acids converted by a conversion factor (Greenfield and Southgate 2003), ^c^raw camelina seed, ^d^boiled camelina seed, ^e^micronized camelina seed, ^f^microwave roasted camelina seed, ^g^raw sunflower seed, ^h^boiled sunflower seed, ^i^micronized sunflower seed, ^j^microwave roasted sunflower seed, ^k^raw flax seed, ^l^boiled flax seed, ^m^micronized flax seed, ^n^microwave roasted flax seed, female/male (usda 2015), ^p^Food Standards for the Polish Population (Jarosz 2012), ^r^intake in British and American diets, ^s^European Food Safety Authority (EFSA 2010), ^t^International Society for the Study of Fatty Acids and Lipids (ISSFAL 2004), ^u^intake in European diets (Mozaffarian et al. 2006), ^w^recommended amount in the diet as prevention of eye diseases (Abdel-Aal et al. 2013), ^z^intake in plant-poor and plant-rich diets (Sikora et al. 2008)

The oil seed processing treatments, in particular boiling (B), resulted in degradation of basic nutrients, e.g. protein, fat, and fiber (6.9–5.7% Cs-B, 27–23% Ha-B, and 2.5–2% Ha-B of daily intake for females/males, respectively). Simultaneously, the level of carbohydrates increased up to 8% of the minimum daily intake (Ha-MWR), which was associated with the modification of the basic chemical composition induced by the infrared (IR) and microwave (MWR) irradiation procedures (Elleuch et al. 2011).

The culinary treatment of the seeds caused an apparent reduction of the fat content and their calorific value even up to 12.6/10.5% (female/male) of the daily intake (Ha-B), but it concurrently increased the SFA level to approximately 12.8/10.4% (female/male) of the daily intake (Ha-IR). Additionally, *trans* isomers of fatty acids, which should not be present in food, were formed in the seeds (Ferreri et al. 2002). The fat fraction composition exhibited a nutritionally adverse decrease in 6n-PUFA and in the particularly valuable 3n-PUFA with ALA.

The camelina and flax seeds exhibited the highest stability of the content of tocopherols and xanthophyll in the raw and processed seeds. However, Dhuique-Mayer et al. (2016) have reported that culinary cooking practices can probably increase the bioavailability of carotenoids mainly by their diffusion to the oil phase, facilitating in vivo transfer thereof into micelles. In turn, phenolics in plant food are very sensitive to thermal treatments (Beta and Hwang 2018). All analyzed oil seed processing treatments resulted in degradation of phenolics. The reduction in the daily intake of these compounds in diets with a varied proportion of plants was estimated on average at 142% (Cs-B, Cs-MWR), 613% (Ha-B), and 14.6% (Lu-IR) (Sikora et al. 2008).

## Conclusion

The analyzed oil seeds are a good source of protein and PUFAs as well as α-tocopherols (Ha-R) and γ-tocopherols (Cs-R, Lu-R), xanthophyll, and phenolics. However, the estimated values of the intake of the selected nutrients and energy suggest that consumption of these seeds should be limited to one portion a day to prevent an intake of excess calories in the usual diet. This amount provides sufficient levels of health-enhancing 3n-PUFA, ALA, tocopherols, and phenolics. The boiling, infrared radiation, and microwave roasting treatments led to loss of crude protein, crude fiber, available carbohydrates, and energy in the camelina and sunflower seeds as well as crude fiber and available carbohydrates in the flax seeds. The most pronounced changes in the fat fraction were induced by micronization and microwave roasting. Nevertheless, despite the modifications of their chemical composition induced by the culinary processing, the analyzed seeds still seem to be a good source of bioactive constituents in the diet.
